# Inverted electro-mechanical behaviour induced by the irreversible domain configuration transformation in (K,Na)NbO_3_-based ceramics

**DOI:** 10.1038/srep22053

**Published:** 2016-02-26

**Authors:** Yu Huan, Xiaohui Wang, Jurij Koruza, Ke Wang, Kyle G. Webber, Yanan Hao, Longtu Li

**Affiliations:** 1 State Key Laboratory of New Ceramics and Fine Processing, School of Materials Science and Engineering, Tsinghua University, Beijing 100084, China; 2 Institute of Materials Science, Technische Universität Darmstadt, 64287 Darmstadt, Germany; 3 Department of Materials Science, Friedrich-Alexander-Universität Erlangen-Nürnberg, 91058 Erlangen, Germany

## Abstract

Miniaturization of domains to the nanometer scale has been previously reported in many piezoelectrics with two-phase coexistence. Despite the observation of nanoscale domain configuration near the polymorphic phase transition (PPT) regionin virgin (K_0.5_Na_0.5_)NbO_3_ (KNN) based ceramics, it remains unclear how this domain state responds to external loads and influences the macroscopic electro-mechanical properties. To this end, the electric-field-induced and stress-induced strain curves of KNN-based ceramics over a wide compositional range across PPT were characterized. It was found that the coercive field of the virgin samples was highest in PPT region, which was related to the inhibited domain wall motion due to the presence of nanodomains. However, the coercive field was found to be the lowest in the PPT region after electrical poling. This was related to the irreversible transformation of the nanodomains into micron-sized domains during the poling process. With the similar micron-sized domain configuration for all poled ceramics, the domains in the PPT region move more easily due to the additional polarization vectors. The results demonstrate that the poling process can give rise to the irreversible domain configuration transformation and then account for the inverted macroscopic piezoelectricity in the PPT region of KNN-based ceramics.

Recently, (K_0.5_Na_0.5_)NbO_3_ (KNN)-based lead-free piezoceramics have attracted considerable attention due to their high Curie temperature, good temperature stability of the piezoelectric properties, and low density. In particular, (Li, Ta, Sb)-modified textured KNN (KNN-LTS) ceramics have been reported to have piezoelectric properties (*d*_33_ ≈ 416 pC/N) comparable to those of some commercial PZT ceramics^1^,^2^. The enhanced piezoelectric properties were initially believed to originate from a morphotropic phase boundary (MPB), similar to the one observed in their well-established lead-based counterparts^3^,^4^. However, it was later found that the property enhancement was not due to a classical MPB, but a polymorphic phase transition (PPT) between the tetragonal and orthorhombic phases^5^,^6^. Even so, domains were found to miniaturize down to the nanometer scale in the two-phase coexistence region of the KNN-based ceramics^7^,^8^,^9^, in a similar manner to that observed in the MPB region of many other piezoelectric materials^10^,^11^,^12^,^13^.

As compared to the micron-sized domains in the single-phase region, the nanodomains have different response mechanisms to the external loads. A lower-symmetry monoclinic structure was detected in the lead-based and lead-free materials with MPB composition, such as Pb(Zr_1−x_Ti_x_)O_3_^10^,^14^,^15^, Pb(Mg_1/3_Nb_2/3_)O_3_-PbTiO_3_^11^, Pb(Zn_1/3_Nb_2/3_)O_3_-PbTiO_3_^16^ and (1−x)(Bi_1/2_Na_1/2_)TiO_3_-xBaTiO_3_^17^,^18^ materials. The monoclinic phase was considered to serve as a structural bridge between rhombohedral and tetragonal phases and facilitate symmetry-allowed polarization rotation. Therefore, the nanodomains are more mobile under an applied electric field, which leads to the enhanced piezoelectric properties near the MPB region^19^,^20^,^21^,^22^. The lower-symmetry monoclinic structure was not detected in the PPT-type piezoelectric materials, such as BaTiO_3_- and KNN-based ceramics. In (1−x)Ba(Zr_0.2_Ti_0.8_)O_3_-x(Ba_0.7_Ca_0.3_)TiO_3_ (BZT-xBCT) ceramics, the ferroelectric phases converge near the line of Curie temperatures. The anisotropic energy contribution in the phase convergence region reduces sharply and even vanishes at a certain composition, as estimated by phenomenological theory^23^,^24^. Thus, nanodomains in the polymorphic phase boundary can respond actively to external loads and lead to the enhancement of piezoelectric properties^25^,^26^. However, orthorhombic and tetragonal phases do not converge near the line of Curie temperatures, as can be seen from the NaNbO_3_-KNbO_3_ phase diagram^27^. Such a situation will result in a larger anisotropic energy contribution along the whole polymorphic boundary line compared with the phase convergence region in BZT-xBCT system^23^. Thus nanodomains in the KNN-based materials may have different behaviours during the application of external loads.

A miniaturized nanoscale domain morphology was previously observed in the virgin state of KNN-based ceramics with two-phase coexistence and was believed to be responsible for the enhanced quasistatic piezoelectric coefficient of the poled ceramics^8^,^9^. However, recent *in situ* transmission electron microscopy (TEM) on bulk KNN-based ceramics revealed that the domain morphology changed significantly during poling process, leading to the noticeable variation of macroscopic piezoelectricity^28^. Therefore, the domain response to external loads in the virgin state can not account for the piezoelectric properties of the poled ceramics. In order to investigate the domain behaviors, the macroscopic electric-field-induced and stress-induced strain curves of the virgin and poled KNN-LTS samples were measured. The observed variations of the electro-mechanical properties before and after the poling process were related to the changes of domain configuration.

## Results

**Figure 1:**
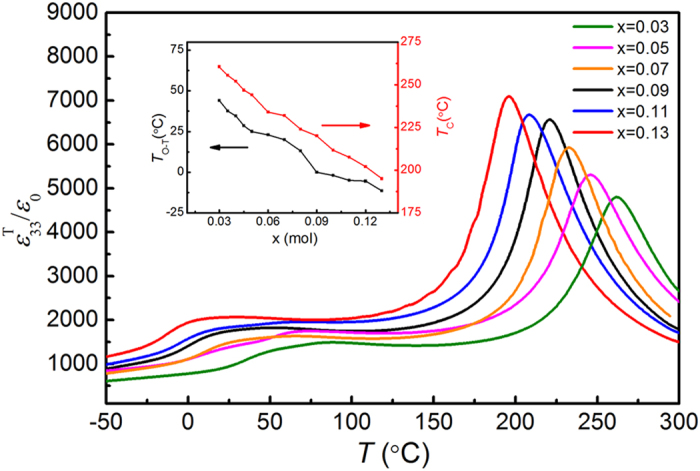
Temperature-dependent 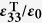 at 1kHz of the poled KNN-LTS ceramics with different Ta molar contents. Inset shows the compositional dependences of *T*_C_ and *T*_O–T_.

Temperature dependence of the relative dielectric permittivity (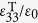) for the poled (Na_0.52_K_0.4425_Li_0.0375_)(Nb_0.92-x_Ta_x_Sb_0.08_)O_3_ (abbreviated KNN-LTS; where x denotes the molar content of Ta) ceramics measured at a frequency of 1 kHz during heating with a rate of 2 °C/min is shown in Fig. 1. The peak at the lower temperature corresponds to the orthorhombic-tetragonal phase transition (*T*_O–T_), while the one at higher temperature to the tetragonal-cubic transition (Curie temperature, *T*_C_). The compositional dependences of *T*_C_ and *T*_O–T_ are summarized in the inset of Fig. 1. The *T*_C_ and *T*_O–T_ decrease almost linearly with increasing Ta content and *T*_C_ lies above room temperature for all investigated compositions. While the ceramics with x < 0.07 have an orthorhombic structure at room temperature, the crystal structure changes to tetragonal for the specimens with x > 0.07. A two-phase coexistence zone composed of orthorhombic and tetragonal ferroelectric phases is formed at room temperature for the composition with x = 0.07. The x = 0.03 specimen is found to have an orthorhombic structure at room temperature, a two-phase coexistence region at about 50 °C, and a tetragonal structure above 50 °C up to 150 °C, as determined by *in-situ* X-ray diffraction (XRD) measurement (Figs S1 and S2).

**Figure 2:**
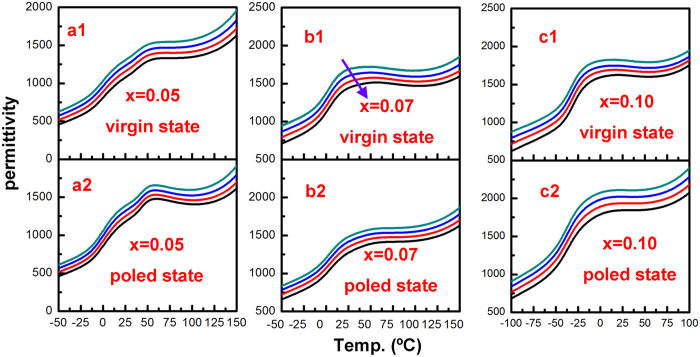
Temperature-dependent dielectric permittivity of the virgin and poled samples measured at frequencies of 0.1, 1, 10, 100 kHz from top.

Temperature dependence of the relative dielectric permittivity for the KNN-LTS poled and virgin samples ceramics measured at frequencies of 0.1, 1, 10, 100 kHz during heating with a rate of 2 °C/min is shown in Fig. 2. “Virgin samples” means that the sintered and machined samples were annealed at 400 °C for 2 h with a cooling rate of 1 °C/min prior to measurement to eliminate the effect of the external factors (stress or electric field) on the domain configuration. No significant frequency dispersion was found for all the poled samples and the virgin samples with x = 0.05 (orthorhombic phase) and x = 0.10 (tetragonal phase). The frequency dispersion was evident for the virgin with x = 0.07 (orthorhombic and tetragonal phase coexistence) near *T*_O–T_ as shown in Fig. 2b1.

The relative density, quasistatic piezoelectric coefficient *d*_33_, planar electromechanical coupling factor *k*_p_, and dielectric permittivity 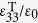 and dielectric loss tan*δ* at 1 kHz of the poled samples with different Ta contents were summarized in Table 1. The relative density of all the samples was relatively high, being more than 96% of the theoretical density. The *d*_33_ and *k*_p_ of the poled ceramics firstly increases and then decreases, reaching a maximum with x = 0.07, which corresponded to the PPT composition. Besides, the specimens have uniform microstructures, as seen from the scanning electron microscope (SEM) images of the KNN-LTS ceramics shown in Fig. S3.

**Figure 3:**
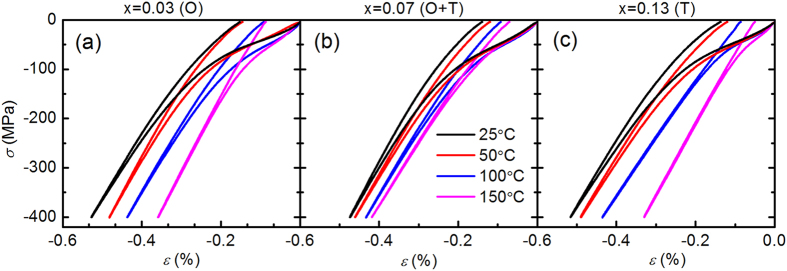
Stress-strain curves for three representative virgin KNN-LTS compositions (O-orthorhombic, T-tetragonal) at four temperatures (25 °C, 50 °C, 100 °C, 150 °C).

**Figure 4:**
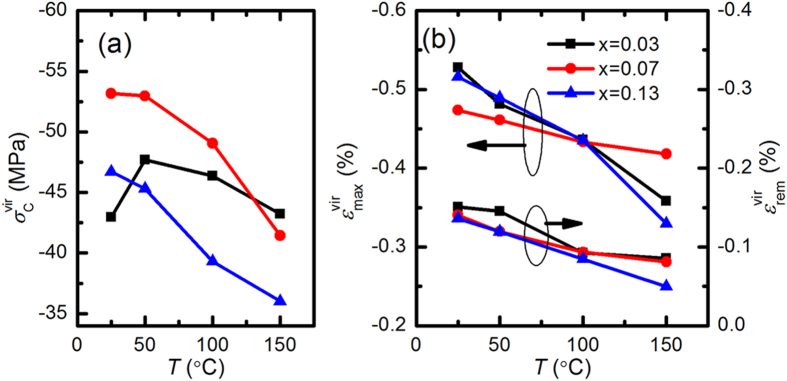
(**a**) Coercive stress 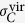, (**b**) mechanically induced maximum strain 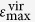 and remanant strain 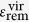 for the three representative virgin KNN-LTS compositions as a function of temperature.

Figure 3 displays the hysteretic stress-strain curves of the virgin samples at various temperatures (25 °C, 50 °C, 100 °C, 150 °C). As expected, all compositions exhibit nonlinear hysteretic ferroelastic behaviour. During loading, the initial stress-strain behaviour is linear elastic. With an increasing applied compressive stress, the stress-strain curve begins to deviate from the linear behaviour as domains begin to ferroelastically reorient. This effect, however, saturates when the amount of switchable ferroelastic domains is exhausted. Therefore, the stress-strain curve again shows linear behaviour at higher stress levels. The characterizing parameters for ferroelastic hysteresis are calculated and illustrated in Fig. 4. Note that the mechanical stress-induced maximum strain (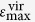) is defined as the strain induced at the maximum compressive stress level of −400 MPa, the remanent strain (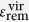) as the plastic strain remaining after mechanical unloading, while the coercive stress (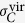) is determined by the inflection point in the loading curve^29^. These parameters were found to depend on the crystal structure, which is influenced by the composition and temperature. The 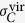 is maximum at 50 °C for the x = 0.03 sample, while it decreases monotonously for the x = 0.07 and x = 0.13 samples upon heating. With increasing the temperature, a corresponding decrease in the 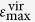 and 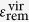 is observed for all compositions.

**Figure 5:**
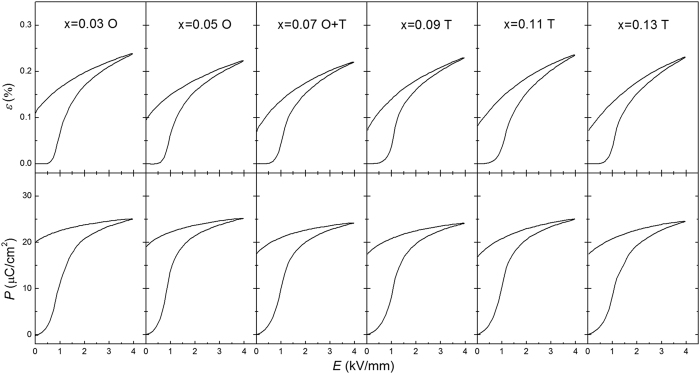
Electric-field-induced strain and polarization hysteresis curves for the virgin KNN-LTS ceramics with different Ta contents at room temperature (O-orthorhombic, T-tetragonal).

**Figure 6:**
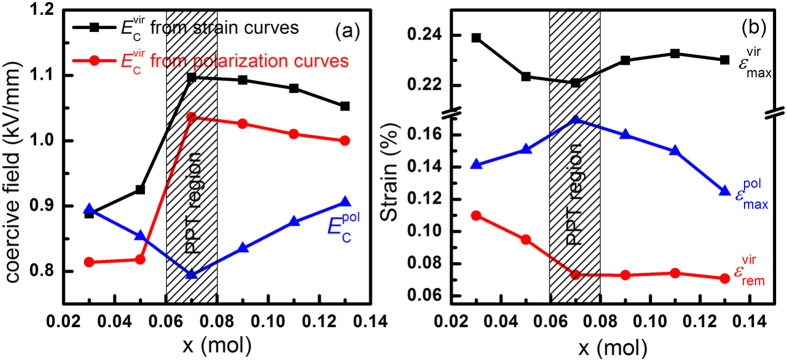
(**a**) Coercive electric field 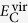 obtained from the unipolar electric-field-induced strain and polarization curves of the virgin KNN-LTS ceramics and coercive electric field 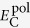 obtained from the bipolar electric-field-induced strain curves of the poled KNN-LTS ceramics. (**b**) Maximum strain 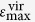 and remanent strain 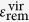 measured during initial unipolar electric field loading of the virgin KNN-LTS ceramics, and maximum strain 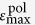 obtained from the unipolar electric-field-induced strain curves of the poled KNN-LTS ceramics as a function of composition.

Figure 5 shows the electric-field-induced strain and polarization curves at a frequency of 1 Hz for the KNN-LTS ceramics at room temperature during the initial electric poling cycle from the virgin state. The electric-field-induced maximum strain (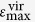) is the strain induced at the maximum electric field of 4 kV/mm, and the remanent strain (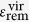) is defined as the remaining strain after removing the applied electric field. The coercive electric field (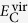) indicates initial domain switching induced by the applied electric field from the virgin state and is determined by the inflection point in the loading curve. Note that some literature reports refer to this field as the “poling field”; however, we avoid this term here to prevent confusion with the field applied during the actual poling process of the ceramics^30^. All of these parameters exhibit a strong dependence on the Ta content, as shown in Fig. 6. The 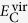, obtained from either strain or polarization curves, reaches a maximum in the proximity of the PPT. With increasing Ta content, the observed electric-field-induced 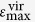 is found to be at a minimum at x = 0.07, which represents the composition with tetragonal and orthorhombic phase coexistence. The electric-field-induced 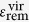 drops rapidly from x = 0.03 to x = 0.07 (orthorhombic phase), but remains almost invariable from x = 0.07 to x = 0.13 (tetragonal phase).

**Figure 7:**
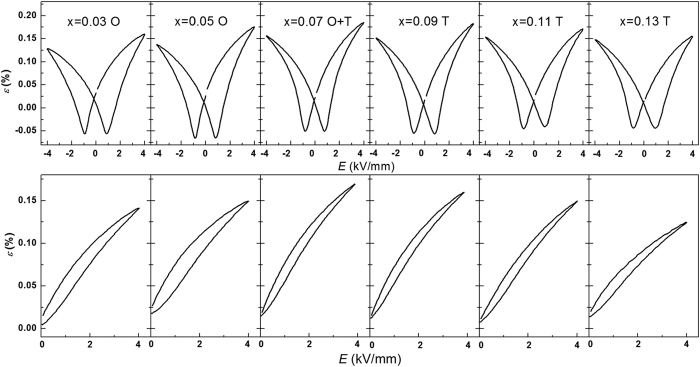
The bipolar and unipolar electric-field-induced strain loops measured at 4 kV/mm for the poled KNN-LTS ceramics at room temperature (O-orthorhombic, T-tetragonal).

Figure 7 shows bipolar and unipolar electric-field-induced strain curves for the poled KNN-LTS compositions, measured at an electric field of 4 kV/mm and a frequency of 1 Hz. The coercive field of poled samples (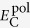) is determined by average value of 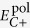 and 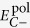 (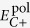 and
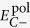
are defined as the electric field where the field-induced strain reaches minimum in the bipolar electric-field-induced curves). The electric-field-induced maximum strain of poled samples (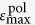) is defined as the strain at peak voltage of the applied electric field according to the unipolar electric-field-induced curves. The 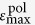 at 4 kV/mm as well as 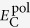 as a function of composition are depicted in Fig. 6. The 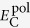 firstly decreases and then increases, reaching a minimum in the proximity of the PPT. Note that this trend is contrary to the trend of 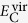 (Fig. 6). The 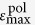
shows an opposite trend as the 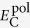. With increasing Ta content, it is found to be at a maximum for x = 0.07, which represents the composition with tetragonal and orthorhombic phase coexistence.

**Figure 8:**
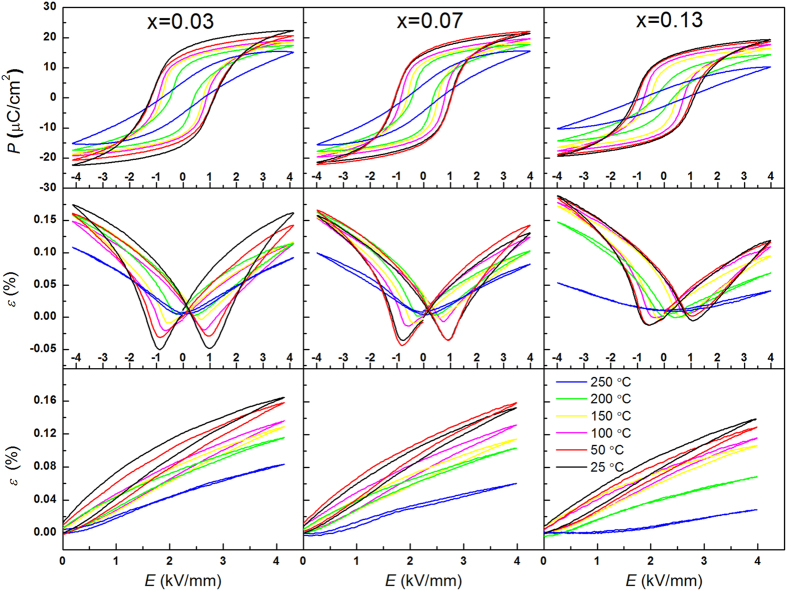
The bipolar polarization and strain loops, and unipolar strain loops at 4 kV/mm of three representative poled KNN-LTS compositions at different temperatures.

**Figure 9:**
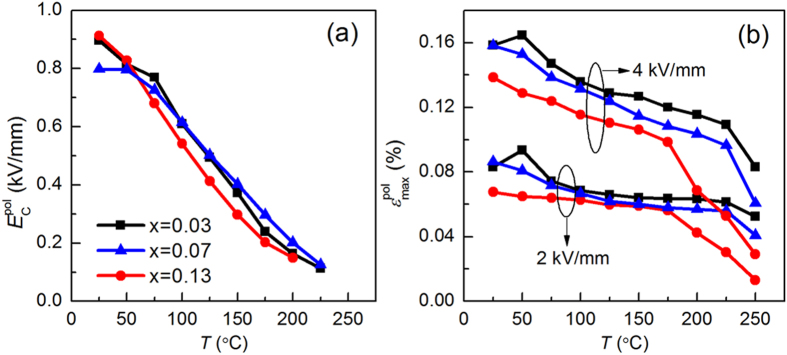
(**a**) Coercive electric field 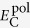 and (**b**) maximum strain 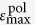
at 2 kV/mm and 4 kV/mm of three representative poled KNN-LTS compositions as a function of temperature.

The bipolar electric-field-induced polarization and strain hysteresis loops, and unipolar electric-field-induced strain curves at 4 kV/mm and 1 Hz for three representative poled KNN-LTS compositions were measured in a broad temperature range, as displayed in Fig. 8. The same electric properties measured at 2 kV/mm are plotted in Fig. S4. Saturated loops are obtained for all samples at temperatures below the *T*_C_, while the rapid decrease of the hysteresis above 200 °C indicates vanishing of the ferroelectric properties. The 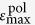 at 2 kV/mm and 4 kV/mm as well as 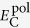 as a function of temperature are depicted in Fig. 9. With increasing temperature, the 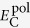
shows a corresponding decrease for all the samples. The 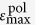 of x = 0.07 and x = 0.13 samples display similar trend as the 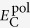, while the 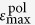 exhibits a peak value at 50 °C for the x = 0.03 sample.

**Figure 10:**
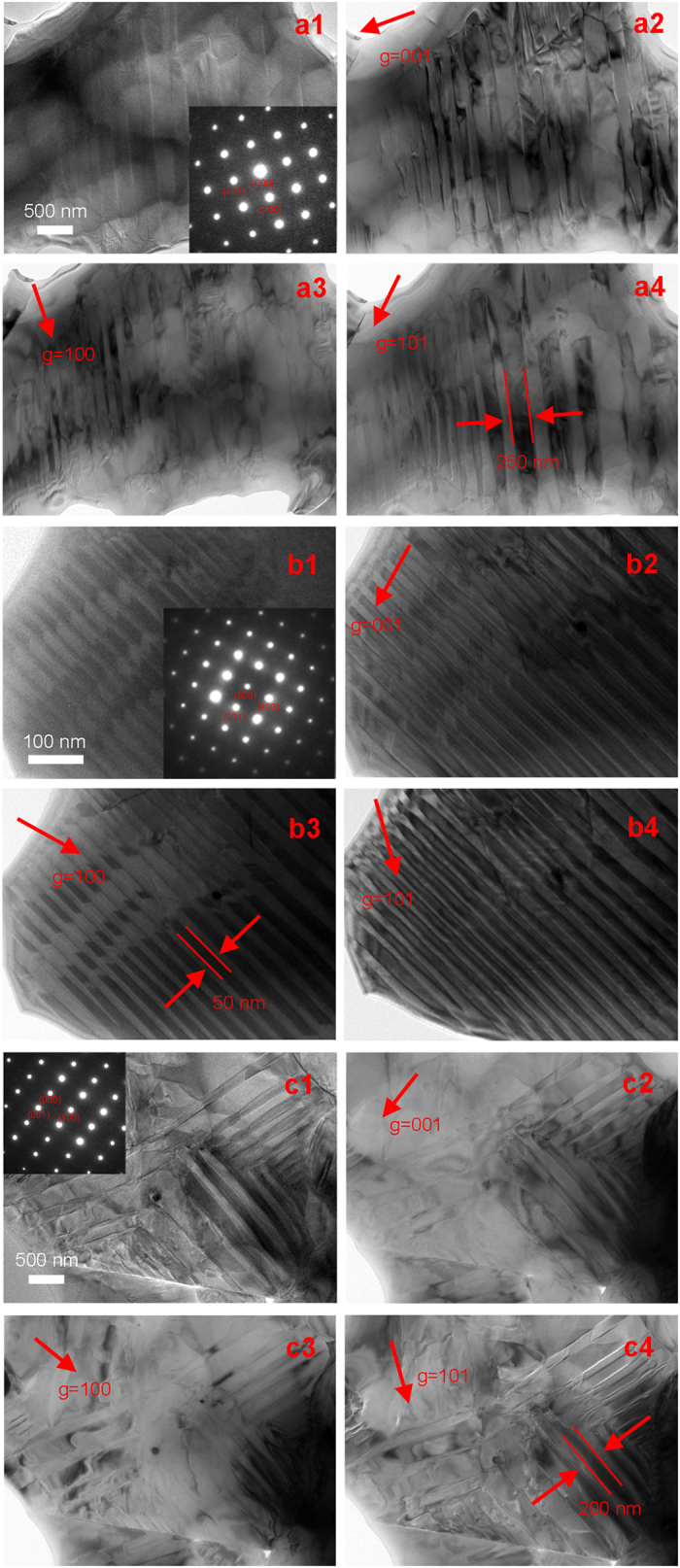
(a1) TEM bright field images of the virgin sample with the composition x = 0.05 along its [010] zone axis with the corresponding diffraction pattern; Observed TEM images under two-beam condition with (a2) g = [001]; (a3) g = [100]; (a4) g = [101]. (b1) TEM bright field images of the virgin sample with the composition x = 0.07 along its [010] zone axis with the corresponding diffraction pattern; Observed TEM images under two-beam condition with (b2) g = [001]; (b3) g = [100]; (b4) g = [101]. (c1) TEM bright field images of the virgin sample with the composition x = 0.10 along its [010] zone axis with the corresponding diffraction pattern; Observed TEM images under two-beam condition with (c2) g = [001]; (c3) g = [100]; (c4) g = [101].

Room temperature TEM bright field images of the virgin KNN-LTS ceramics are shown in Fig. 10. Distinct changes in the domain size and morphology are observed with increasing x. Micron-sized lamellar domain structure is dominant in the sample with x = 0.05 exhibiting a pure orthorhombic phase (in Fig. 10a1). On the other hand, typical 90° domain morphology with parallel stripes is observed in the purely tetragonal samples with x = 0.10 (in Fig. 10c1). Stripe and herringbone domains were previously reported to be a typical feature of domain configuration in KNN-based ceramics with a tetragonal and orthorhombic phase^31^,^32^. The TEM bright field images under different two-beam condition were taken to verify that nanosacle domain did not exist in x = 0.05 and x = 0.10 specimens. The average domain width observed in the virgin ceramic with pure tetragonal and pure orthorhombic phase was micron scale. The domain width minimized down to nanoscale in the proximity of the PPT as schematically shown in Fig. 10b1. We took a series of TEM images under different two-beam condition and found the domain width was about 50 nm. In addition, these nanodomains are near-periodically spaced and have a stripe-like morphology. Nanoscale domain morphology near the PPT region can relieve the internal stress associated with phase transition, which has been found by previous studies^8^,^9^.

## Discussion

The electric-field-and stress-induced strain curves from the virgin state were measured in order to study the domain behaviour of the virgin KNN-LTS ceramics under the external loads. The coercive field is directly related to the domain switching possibility during the application of an external load^33^,^34^. As shown in Fig. 6, the 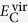
obtained from the virgin strain curves increases with compositional proximity to the PPT. Correspondingly, the 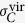 obtained from virgin stress-strain curves at room temperature is higher for the x = 0.07 sample than for x = 0.03 and x = 0.12 samples (Fig. 4a). Both parameters demonstrate that the domains of virgin samples are more difficult to switch when orthorhombic and tetragonal phases coexist, as compared to samples with pure tetragonal or orthorhombic phases. In addition, as evident from the temperature-dependent stress-strain curves in Fig. 4a, the x = 0.07 and x = 0.13 samples exhibit a continued reduction in 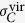 with increasing temperature since the domain walls move more easily at higher temperature. However, the 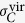 of the x = 0.03 specimen firstly increases and then decreases, reaching a maximum near the orthorhombic-tetragonal phase transition point at 50 °C. The 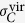 at 50 °C is higher than at 25 °C demonstrating that the domain configuration is in a more stable condition at 50 °C (near PPT region) than at 25 °C. Therefore, the 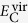 and 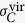 of the virgin KNN-LTS ceramics reach maximum in the proximity of PPT region, which indicates that the ferroelectric and ferroelastic domains are more difficult to switch when two phases coexist.

On the other hand, the electric-field- and stress-induced 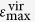 of x = 0.07 sample at room temperature are lower than that of x = 0.03 and x = 0.12 samples, as shown in Figs 4b and 6b. The temperature-dependent stress-strain curves display that the stress-induced 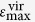 of the x = 0.07 and x = 0.13 specimens decreases nearly linearly from 25 °C to 100 °C. However, rate of decrease with increasing temperature for the x = 0.03 sample is 1.8∙10^−3^%∙°C^−1^ from 25 °C to 50 °C and 0.9∙10^−3^%∙°C^−1^ from 50 °C to 100 °C, respectively. The sharp reduction of stress-induced 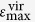 at 50 °C (orthorhombic-tetragonal phase transition point) demonstrates that the domain mobility is restricted in the PPT region. Therefore, minimization of the electric-field- and stress-induced 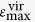 in the PPT region also confirms that the domain mobility is constrained when two phases coexist, corresponding to the results of 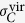 and 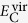.

**Figure 11:**
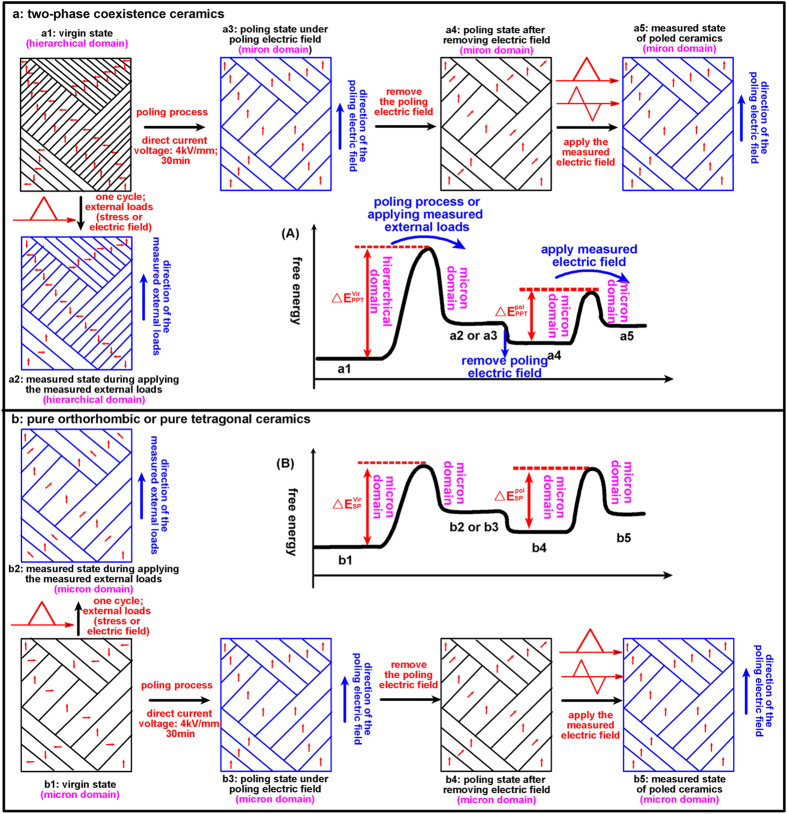
Schematic diagram of domain configuration for the two-phase coexistence ceramics (**a**) and for the pure orthorhombic or pure tetragonal ceramics (**b**) at different states; Insets show the conceptual energy landscape of different states for the two-phase coexistence ceramics (A) and for the single-phase ceramics (B).

This domain behaviour in the virgin samples can be elucidated from the view of the domain configuration. The evolution of the domain configuration for two-phase coexistence ceramics and the pure orthorhombic or pure tetragonal ceramics is displayed in the schematic diagrams of Fig. 11a,b, respectively, while the corresponding conceptual free energy landscape is plotted as inset in Fig. 11A,B. As evident from TEM images in Fig. 10, the nanoscale domain structure exists in the virgin ceramics with two-phase coexistence (Fig. 11a1), while the micron-sized lamellar and herringbone domain structures are dominant in virgin ceramics with pure tetragonal or pure orthorhombic phases (Fig. 11b1). Under the application of external loads, the domain walls move and the spontaneous polarization switches, as shown in Fig. 11a2,b2^35^. In this process, the energy barrier (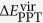 or 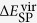) must be overcome. On one hand, the anisotropic energy term between orthorhombic and tetragonal polarization states near the PPT region does not vanish according to phenomenological theory^23^. Therefore, the domain walls would act as a barrier to the domain translation and switching because of the energy barrier for polarization rotation, which is different from the vanishing anisotropy energy and drastic decreasing domain wall energy near the MPB region^36^ or in the phase convergence region of the piezoelectric systems^3^. The miniaturized domain in the PPT region will increase the domain wall density, leading to a larger energy barrier 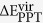 in the PPT region compared with 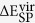 in the ceramics with single phase. On the other hand, the domain wall will impede each other. Hence domain wall motion is more difficult in the PPT region than that in the single-phase region with micron-sized domain structures. The energy barrier 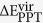 of the ceramics with two-phase coexistence is larger than 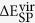 of the ceramics with pure tetragonal or pure orthorhombic phase. Therefore, the 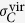 and 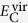 reach maximum in the proximity of the PPT region. Correspondingly, the electric-field- and stress-induced 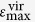 have a minimum value in the PPT region.

The electrical properties and domain behaviour of the poled samples are different from that of the virgin samples. The 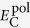 obtained from the bipolar strain curves of the poled samples at room temperature minimizes in the proximity of PPT region (Fig. 6a). As evident from the temperature-dependent bipolar strain curves in Fig. 8, the 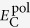 decreases with increasing temperature due to the lowering of the activation energy for domain reorientation. It decreases linearly in the pure tetragonal region, while it shows a nonlinear tendency across the PPT region for all the three compositions as shown in Fig. 9a. Specifically, the 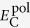 did not significantly decline from 25 °C to 50 °C for x = 0.07 sample. Also, the rate of decrease for x = 0.03 sample was 3.3∙10^−3^ kV/mm∙°C^−1^ from 25 °C to 50 °C and 1.8∙10^−3^ kV/mm∙°C^−1^ from 50 °C to 75 °C, respectively. These results illustrate that the 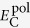 decreases sharply with increasing temperature proximity to PPT region. Therefore, the 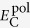 of the poled samples has a minimum in the PPT region, indicating easier domains switching. With increasing Ta content, the 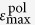 obtained from the unipolar strain curves at room temperature is found to have a peak value in x = 0.07, which represents the composition with tetragonal and orthorhombic phase coexistence. With increasing temperature the 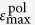 decreases monotonously for x = 0.07 and x = 0.13 samples and has a peak value at 50 °C (orthorhombic-tetragonal phase transition point) for x = 0.03 sample. The maximization of 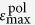 in the PPT region also confirms that the domains are more flexible to move when two ferroelectric phases coexist, which is contrary to the electrical properties and domain behaviour of the virgin samples.

The electro-mechanical properties can be elucidated from the domain behaviour in the poled samples, which is closely related to the domain configuration. In many lead-free and lead-based piezoelectric ceramics, it is verified that regular ferroelectric domains correspond to minimum frequency dispersions in dielectric permittivity while nanodomains lead to strong frequency dispersions^13^,^37^,^38^,^39^. No significant frequency dispersion is found for x = 0.05 and x = 0.10 samples before and after poling process. Hence, the domain size in virgin and poled ceramic with pure tetragonal and orthorhombic phase is micron scale. In the meantime, the dielectric permittivity characterized by a relatively strong frequency dispersion in the virgin ceramics with x = 0.07 near *T*_O–T_ indicates a short range order with random nanodomains, corresponding to the TEM images in Fig. 10b1. But frequency dispersion decreases remarkably and diminishes even after poling process in Fig. 2b2. It means the nanoscale domain configuration (virgin state in Fig. 11a1) of the ceramics with two-phase coexistence can gradually coalesce and assemble into micron-sized domains structure under the high direct current electric field (poling state in Fig. 11a3) in the poling process. Such nanodomain growth under the electric field was previously supported by numerous experimental observations on many lead-based and lead-free piezoelectrics, including Pb(Zr_1−x_Ti_x_)O_3_ based^40^, Pb(Mg_1/3_Nb_2/3_)O_3_-PbTiO_3_ based^41^,^42^, (Bi_1/2_Na_1/2_)TiO_3_-BaTiO_3_ based^43^, and BaTiO_3_ based^44^,^45^ composition. After removing the poling field, the micron-sized domain structure (poling state in Fig. 11a4) is retained. Consequently, all the samples with different crystal structures have similar micron-sized domain configuration after poling process in Fig. 11a4,b4. In addition, 18-fold degenerate domain variants exist in PPT region, while only 12 variants are possible in pure orthorhombic region and 6 variants in pure tetragonal region. Hence, the domains reorient more easily in the PPT region as compared to pure tetragonal or pure orthorhombic regions when an electric field is applied to the poled ceramics. The energy barrier 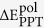 of the ceramics with two-phase coexistence is smaller than 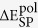 of the ceramics with single phase. Therefore, the 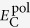 of the poled samples reach minimum for the PPT composition and 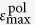 has a peak value in the PPT region.

## Conclusions

The Li, Ta, Sb-modified KNN ceramic samples with different Ta contents were prepared using the conventional mixed-oxide method and the electro-mechanical properties were investigated. A significant variation in the electrical and mechanical properties was observed before and after the poling process. The highest coercive field and lowest field-induced strain were obtained in the virgin samples with PPT composition, while the opposite trend was detected after the ceramics were poled. The electro-mechanical properties could be elucidated from the differences in the domain configuration. In the virgin samples, the micron-sized domain structures are dominant in the pure tetragonal or pure orthorhombic ceramics, while nanoscale domain structures exist in the two-phase coexistence ceramics. The miniaturized domain induced high domain wall density leads to a larger domain wall energy barrier in the PPT region, making the domain wall mobility difficult. Therefore, the coercive field of the virgin samples reaches the maximum in the proximity of the PPT region. During the poling process, the nanodomains transform irreversibly into micron-sized domain structure and thus all the poled KNN-LTS ceramics have similar micron-sized domain configuration. The domains in the PPT region move more easily due to the additional polarization vectors. Therefore, the coercive field of the poled ceramics has a minimum value for the PPT composition.

## Methods

(Na_0.52_K_0.4425_Li_0.0375_)(Nb_0.92−x_Ta_x_Sb_0.08_)O_3_ (with x = 0.03, 0.035, 0.04, 0.045, 0.05, 0.06, 0.07, 0.08, 0.09, 0.10, 0.11, 0.12, 0.13) powders were prepared using the conventional mix-oxide method. Analytically pure powders of Nb_2_O_5_ (Sinopharm Chemical Reagent Beijing Co., Ltd, 99.5% purity), Ta_2_O_5_ (Aladdin, 99.9% purity), Sb_2_O_3_ (Aladdin, 99.9% purity), Na_2_CO_3_ (Sinopharm Chemical Reagent Beijing Co., Ltd, 99.8% purity), K_2_CO_3_ (Sinopharm Chemical Reagent Beijing Co., Ltd, 99% purity), and Li_2_CO_3_ (Sinopharm Chemical Reagent Beijing Co., Ltd, 99% purity) were utilized as starting materials. The alkali carbonates were first dried at 80 °C and then homogenized with other oxides in a planetary mill for 8 h according to the nominal stoichiometric composition using ethyl alcohol as medium. The powder mixtures were calcined twice at 850 °C for 5 h with intermediate milling. Subsequently, they were milled, dried, and sieved. The powders were then compacted into pellets with a diameter of 10 mm and a thickness of 1 mm by uniaxial pressing in a stainless-steel die using Polyvinyl Butyral as binder. The specimens were sintered at 1090 °C for 2 h with a heating/cooling rate of 3 °C/min in a sealed crucible to minimize the evaporation of alkaline metals. The density of the samples was determined by the Archimedes method. Scanning electron microscopy (SEM; Leo-1530, Oberkochen, Germany) was used to examine the microstructure of the sintered ceramics. The crystalline structure of the crushed sintered ceramics was determined by XRD (Rigaku 2500, Rigaku, Tokyo, Japan) with Cu Kα radiation. After mechanical polishing and ion milling of ceramics, domain structure was studied using the TEM, carried out on electron microscope TECNAI G^2^ 20 (FEI, Hillsboro, OR, USA) at 200 kV.

The KNN-LTS powders with the composition of x = 0.03, 0.07, 0.13 were compacted into cylinder with a diameter of 10 mm and a thickness of 7 mm and then sintered at 1110 °C for 2 h. The sintering temperature of the cylindrical samples was slightly higher than the discoid samples in order to obtain the same density and microstructure. Cylindrical shaped samples with a diameter of ∼5.8 mm and a height of ∼6 mm were obtained by core drilling. Each sample was annealed at 400 °C for 2h with a heating and cooling rate of 5 °C/min and 1 °C/min, respectively, prior to mechanical testing to eliminate residual stresses, which may be induced by the machining process. To characterize the ferroelastic behaviour, the stress-strain curves were measured with an experimental setup described in detail elsewhere^46^. Mechanical compressive stress up to -400 MPa with a loading rate of 4 MPa/s was applied to the preloaded specimen, centered by an alumina alignment fixture. After reaching the maximum compressive stress, the sample was unloaded with the same rate back to the preload stress. The stress-induced uniaxial displacement of the specimen was measured by a linear variable differential transducer (LVDT). The experimental error was previously determined as ±2% for both maximum and remanent strain. The measurement temperatures in this study ranged from room temperature up to 150 °C.

For electric measurement, the two main surfaces of the sintered disk samples were coated with silver paste and then heat-treated at 550 °C for 30 min with a cooling rate lower than 1 °C/min to reduce internal stress. During measurement a unipolar triangular electrical load was applied to the sample at a frequency of 1 Hz using a high voltage amplifier (Trek Model 20/20C, TEGAM, Cleveland, OH, USA) and a function generator (Agilent 33220A, Agilent, Santa Clara, CA, USA). The strain and polarization were measured during testing with a LVDT and a Sawyer-Tower circuit, respectively. The disk samples were poled in silicon oil under a direct current electric field of 4 kV/mm at 70 °C for 30 min. The quasistatic piezoelectric coefficient *d*_33_ of the poled samples was measured using a quasistatic *d*_33_ meter (ZJ-3A, Insitute of Acoustics, Chinese Academy of Sciences, Beijing, China). Dielectric permittivity 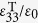, dielectric loss tan*δ* at 1 kHz and the planar electromechanical coupling factor *k*_p_ at room temperature were measured using a capacitance meter (Agilent 4294A, Agilent, Santa Clara, CA, USA). The temperature-dependent dielectric properties of the poled and virgin samples were measured from −50 °C to 300 °C with a heating rate of 2 °C/min (Alpha-A High Performance Frequency Analyzer equipped with a cryostat, Novocontrol Technologies, Montabaur, Germany). The electric field-polarization and electric field-strain curves were measured from room temperature to 250 °C by using the TF ANALYZER 2000E ferroelectric measuring system (aixACCT Systems GmbH, Aachen, Germany).

## Additional Information

**How to cite this article**: Huan, Y. *et al*. Inverted electro-mechanical behaviour induced by the irreversible domain configuration transformation in (K,Na)NbO_3_-based ceramics. *Sci. Rep*. **6**, 22053; doi: 10.1038/srep22053 (2016).

## Supplementary Material

Supplementary Information

## Figures and Tables

**Table 1:** Variations of the relative density, *d*_33_, *k*_p_, 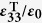 , and tan*δ* of the poled KNN-LTS ceramics with different Ta contents at room temperature.

Ta content (x)	0.03	0.035	0.04	0.045	0.05	0.06	0.07	0.08	0.09	0.1	0.11	0.12	0.13
Relative density (%)	96.5	97.0	98.2	98.5	98.4	98.2	98.1	98.5	99.1	98.7	98.2	97.5	98.2
*d*_33_ (pC/N)	232	240	248	255	263	296	321	318	297	286	250	232	215
*k*_p_ (%)	36	40	39	42	41	46	47	46	44	43	39	36	33
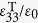	1289	1318	1400	1436	1501	1605	1748	1822	1883	1919	1979	1993	2013
tan_*δ*_	0.030	0.032	0.027	0.032	0.033	0.027	0.027	0.022	0.028	0.021	0.028	0.028	0.027
