# Supplementary material for: Inverted electro-mechanical behaviour induced by the irreversible domain configuration transformation in (K,Na)NbO3-based ceramics
Source: Sci Rep. 2016 Feb 26;6:22053. doi: 10.1038/srep22053 (PMC4768104; doi:10.1038/srep22053)
Supplement: Supplementary Information [file srep22053-s1.doc]

**Supplementary information**

**Inverted** **electro-mechanical behaviour induced by the** **irreversible** **domain configuration transformation in (K,Na)NbO_3_-based ceramics**

Yu Huan^1^, Xiaohui Wang^1,*^, Jurij Koruza^2^, Ke Wang^1^, Kyle G. Webber^3^ Yanan Hao^1^ & Longtu Li^1^

^1^State Key Laboratory of New Ceramics and Fine Processing, School of Materials Science and Engineering, Tsinghua University, Beijing 100084, China

^2^Institute of Materials Science, Technical University Darmstadt, 64287 Darmstadt, Germany

^3^Department of Materials Science, Friedrich-Alexander-Universität Erlangen-Nürnberg, 91058 Erlangen, Germany

^*^Correspondence and requests for materials should be addressed to Xiaohui Wang ([wxh@mail.tsinghua.edu.cn](mailto:wxh@mail.tsinghua.edu.cn))


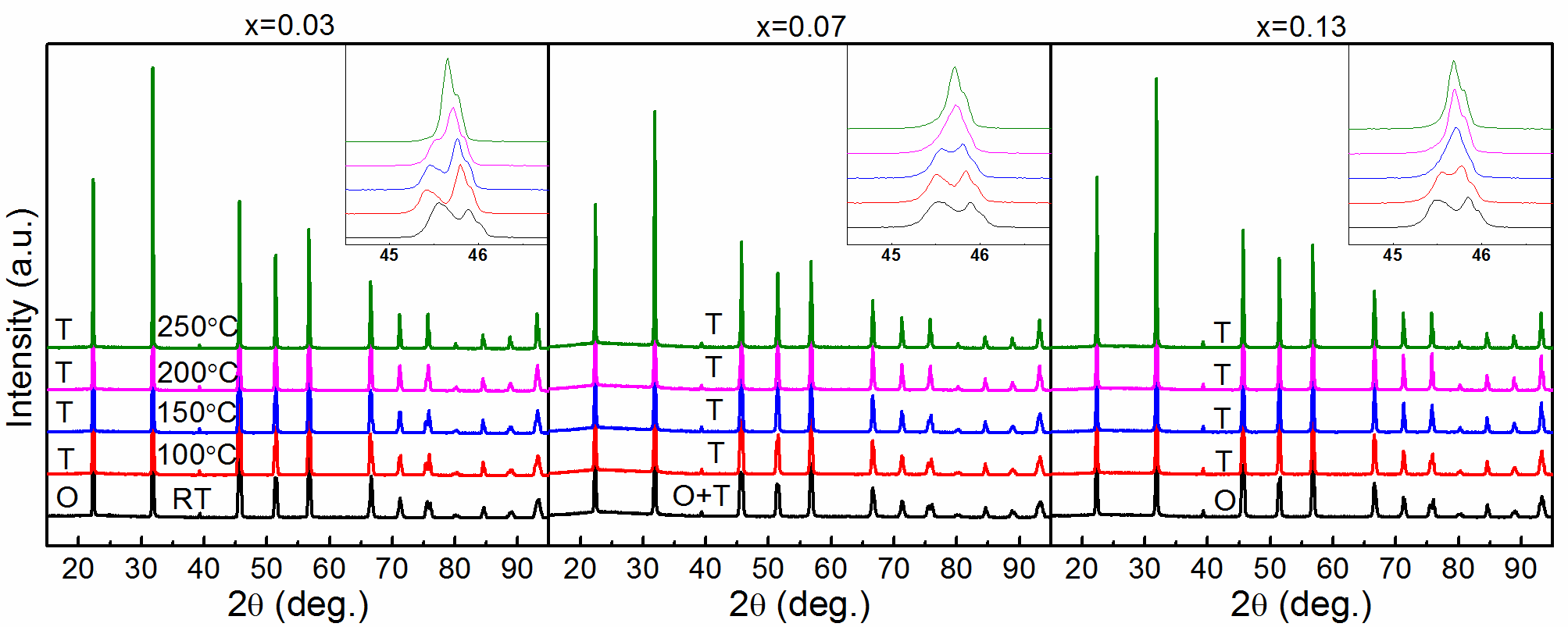


Figure S1 (Color online) XRD patterns of three representative KNN-LTS compositions at different temperatures. Insets are the corresponding expanded XRD patterns in the 2θ ranges from 44.5° to 46.5° (O-orthorhombic, T-tetragonal)

High-temperature XRD was performed at a series of temperature (RT, 100 °C, 150 °C, 200 °C, 250 °C) to identify the phase transitions (Fig. S1). All samples exhibited a pure peroviskite phase structure indicating that diffusion of the doped elements (Li, Sb, and Ta) into the (K_0.5_Na_0.5_)NbO_3_ lattice was complete. The {200} diffraction lines in the 2 range between 44.5° and 46.5° were contrasted in the insets and a pronounced difference in crystal structures can be found as the temperature increased.


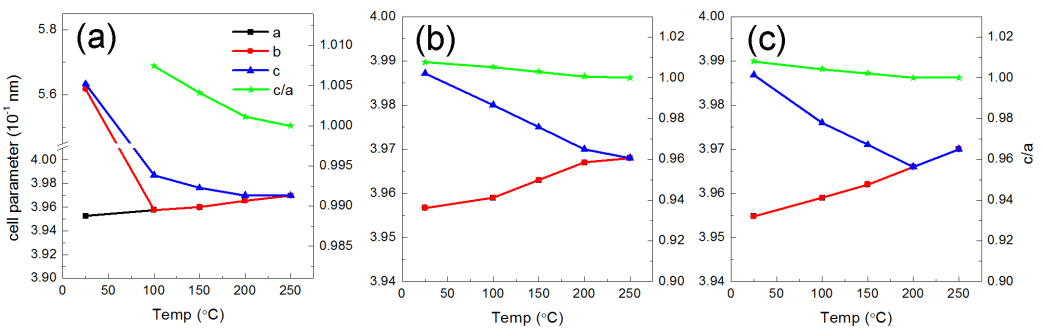


Figure S2 (Color online) The variation of the lattice parameters of three representative KNN-LTS compositions as function of temperature: (a) x = 0.03; (b) x = 0.07; (c) x = 0.13.

The lattice parameters of the ceramics with three representative compositions at different temperatures (Fig. S2) were obtained from the Rietveld analysis, performed using the JANA 2006 software.[^1^](#_ENREF_1) The background was modeled using the Legendre polynomial and the peaks’ profiles were refined using a pseudo-Voigt function. The chosen space groups for these refinements were *Amm*2 (JCPDS-ICDD file no. 71-0946) and *P*4*mm* (JCPDS-ICDD file no. 71-0946) for the orthorhombic and tetragonal KNbO_3_ phases, respectively. The lattice parameters and c/a as a function of temperature are shown in Fig. S2. All the samples, except x = 0.03 and x = 0.07 samples at RT, display a tetragonal structure. The calculated c/a monotonously decreases with increasing temperature. The x = 0.03 specimen was found to have an orthorhombic structure at RT and tetragonal structure from 100 °C to 250 °C. The x = 0.07 displayed the coexistence of tetragonal and orthorhombic phases at RT, and the tetragonal phase became dominant with increasing temperature. The x = 0.13 specimen had a tetragonal structure from RT to 200 °C and became cubic at 250 °C. These results were in agreement with the results obtained by the permittivity versus temperature curves in Fig. 1.


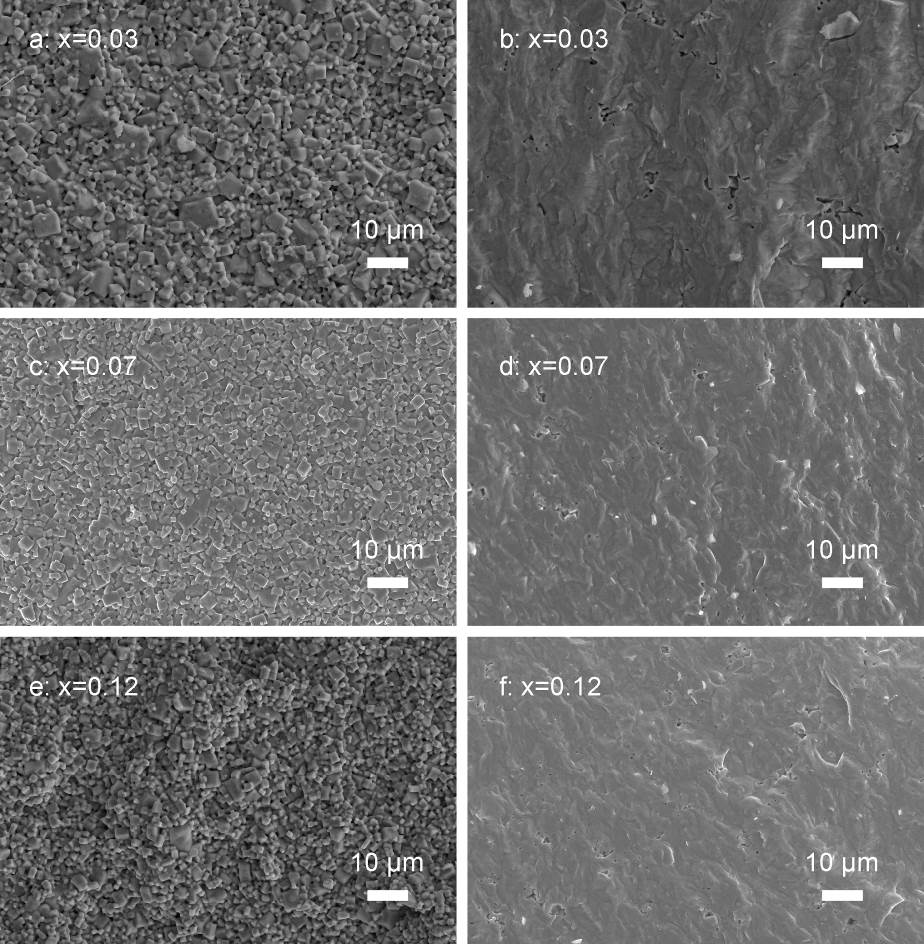


Figure S3 (Color online) SEM images of the KNN-LTS ceramics with different Ta molar contents (a-b) x = 0.03, (c-d) x = 0.07, (e-f) x = 0.12. The left images are the surface of the ceramics, the right images are the fracture surface of the ceramics.

Figure S3 shows the microstructure of the KNN-LTS ceramics with different Ta contents. A characteristic cubic or rectangular morphology of the grains with a clear grain boundary can be seen at the surface of the ceramics. The grain size decreases with increasing Ta content, in agreement with previous reports.[^2^](#_ENREF_2) The microstructure remains relatively uniform in all ceramics. In addition, all samples show predominantly transgranular fracture. Few pores can be seen from the SEM images, which suggests a high density of those samples. The density measured by the Archimedes method is higher than 96 % for all samples.


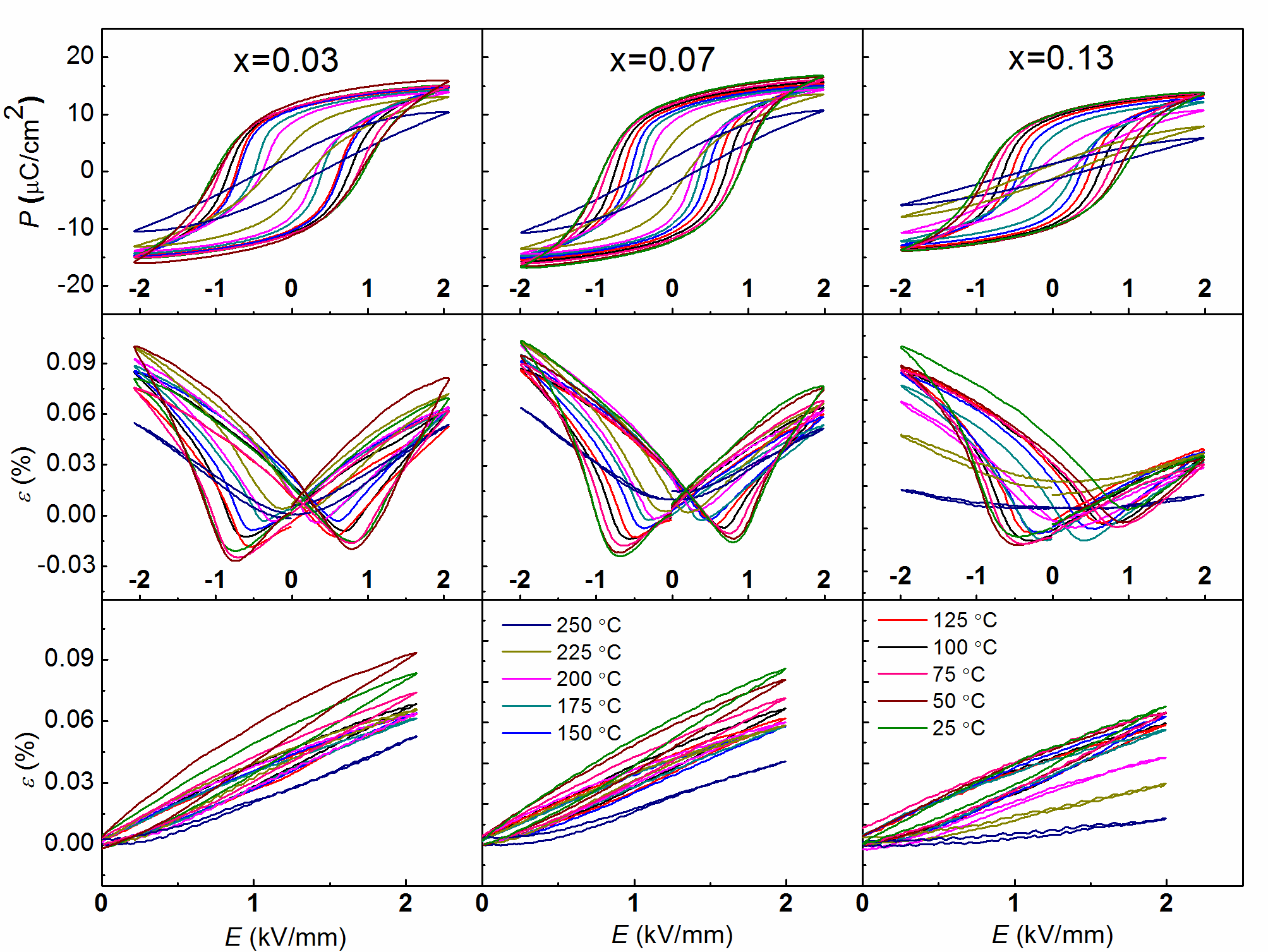


Figure S4 The electric field-polarization loops, bipolar electric field-strain loops and unipolar electric field-strain loops at 2 kV/mm of three representative poled KNN-LTS compositions at ten temperatures (25 °C, 50 °C, 75 °C, 100 °C, 125 °C, 150 °C, 175 °C, 200 °C, 225 °C, 250 °C)

**References:**

1. Petříček, V., Dušek, M. & Palatinus, L. Crystallographic computing system JANA2006: general features. *Z. Kristallogr.* **229**, 345-352 (2014).

2. Yang, Z. P., Chang, Y. F. & Wei, L. L. Phase transitional behavior and electrical properties of lead-free (K_0.44_Na_0.52_Li_0.04_)(Nb_0.96-x_Ta_x_Sb_0.04_)O_3_ piezoelectric ceramics. *Appl. Phys. Lett.* **90**, 042911 (2007).
